# Korean mistletoe (*Viscum album coloratum*) extract regulates gene expression related to muscle atrophy and muscle hypertrophy

**DOI:** 10.1186/s12906-017-1575-9

**Published:** 2017-01-21

**Authors:** Juseong Jeong, Choon-Ho Park, Inbo Kim, Young-Ho Kim, Jae-Min Yoon, Kwang-Soo Kim, Jong-Bae Kim

**Affiliations:** 1 School of Life Science, Handong Global University, Pohang, 37544 Korea; 2 Mistle Biotech Co., Ltd., Pohang, 37668 Korea; 3 Food R&D Center, Samyang Corporation, Incheon, 22826 Korea

## Abstract

**Background:**

Korean mistletoe (*Viscum album coloratum*) is a semi-parasitic plant that grows on various trees and has a diverse range of effects on biological functions, being implicated in having anti-tumor, immunostimulatory, anti-diabetic, and anti-obesity properties. Recently, we also reported that Korean mistletoe extract (KME) improves endurance exercise in mice, suggesting its beneficial roles in enhancing the capacity of skeletal muscle.

**Methods:**

We examined the expression pattern of several genes concerned with muscle physiology in C2C12 myotubes cells to identify whether KME inhibits muscle atrophy or promotes muscle hypertrophy. We also investigated these effects of KME in denervated mice model.

**Results:**

Interestingly, KME induced the mRNA expression of SREBP-1c, PGC-1α, and GLUT4, known positive regulators of muscle hypertrophy, in C2C12 cells. On the contrary, KME reduced the expression of Atrogin-1, which is directly involved in the induction of muscle atrophy. In animal models, KME mitigated the decrease of muscle weight in denervated mice. The expression of Atrogin-1 was also diminished in those mice. Moreover, KME enhanced the grip strength and muscle weight in long-term feeding mice.

**Conclusions:**

Our results suggest that KME has beneficial effects on muscle atrophy and muscle hypertrophy.

## Background

Muscle atrophy is a feature of aging, starvation, cancer, diabetes, muscle denervation, and other physical conditions that limit muscle use [1]. The decrease of muscle amount in the human body is considered as a symptom of the disorder. With the increase of average life expectancy, the number of cancer and diabetes patients has also increased. Thus, the importance of maintaining muscle mass is increasing. Although muscle atrophy occurs frequently, there are no common clinical therapies to prevent or repress the muscle atrophy in human cases to date [2].

Over the last few decades, the mechanisms related to muscle mass decrease have not been clearly elucidated. Many research studies were undertaken to find the molecular mechanisms responsible for muscle mass decrease and increase. However, recent studies strongly support the idea that there are specific signaling pathways regulating the muscle weight increase and decrease [3]. Based on the recent reports, muscle mass regulation is closely related to the PI3K/Akt pathway [4]. In the case of muscle hypertrophy, it is generally known that increasing muscle mass is not because of an increased number of muscle cells but because of an increased amount of intracellular protein [5]. In the case of muscle atrophy, the decreasing muscle mass is the opposite of muscle hypertrophy—decreased proteins in muscle cells [6]. People called the genes that regulate this specific, muscle mass decreasing mechanism Murf1 and Atrogin-1 [7]. These genes are closely linked with protein ubiquitination [8, 9]. Nowadays, the aims of studies regarding muscle atrophy and therapy for muscle diseases are to find the unknown molecules or mechanisms regulating Murf1 and Atrogin-1. Murf1 and Atrogin-1 are members of the E3 ubiquitin ligase family [10, 11]. The targets of these E3 proteins are intimately involved with maintaining the structure of muscle cells and the construction of muscle [12]. It was also proven that these E3 ubiquitin ligases are regulated by the MAPK signaling pathway, including FoxO [13]. These genes stimulating muscle mass decrease are repressed by the MAPK signaling pathway and, are activated by FoxO [14]. In the case of muscle hypertrophy, previous studies verified that the Akt/mTOR signaling pathway is the main signaling pathway that regulates the muscle mass increase [15]. The Akt/mTOR pathway is affiliated with several other intracellular signaling pathways and key molecules for survival, proliferation, stimulation of the cell cycle, and many metabolic regulators [16, 17].

Mistletoe is a semi-parasitic plant that has been utilized as a traditional medicine in many countries to treat various human illnesses [18, 19]. In particular, Korean mistletoe (*Viscum album coloratum*) has been widely studied in the last decades since the report that it has a anticancer activity [20]. It has been reported that Korean mistletoe extract (KME) has a variety of effects on biological functions, demonstrating anti-tumor, anti-oxidant, anti-diabetes, and anti-obesity benefits, and promotes the extension of lifespan [21–23]. In addition, we have recently reported that KME improved the endurance capacity in mice by enhancing mitochondrial activity [24]. In the treadmill and swimming tests, KME-treated mice showed an increased exercise capacity compared to chow-fed control mice. Therefore, it is conceivable that KME also has beneficial functions in enhancing the capacity of skeletal muscle in mice.

In this study, we discovered that KME can induce the phosphorylation of Akt signaling pathway. Therefore, we hypothesized that the increase and decrease of muscle mass can be regulated by KME. Murf1 and Atrogin-1 were chosen as target genes to identify the inhibitory effect of KME in muscle atrophy. We treated mouse myoblast C2C12 cells with KME, and adopted an artificially induced muscle atrophy condition (denervation) [25] in mice to validate its effects in vivo.

## Methods

### Preparation of KME

Korean mistletoe extract (KME) was supplied by Daeho Corporation (Hwasung, Korea). Summarized manufacturing process is as follows. The leaves, twigs, and fruits of Korean Mistletoe plants were homogenized in 7 volumes of distilled water, using a blender. The homogenized Korean Mistletoe extracts were stirred for 8 h at 100 °C. Then, secondary extraction was performed with 4 volumes of distilled hot water and filtered with 1 μm Whatman™ filter paper. After filtration, the mistletoe extract was evaporated in vacuum condition and stored at 4 °C until use.

### Reagents and antibodies

Dulbecco’s modified Eagle’s medium (DMEM), fetal bovine serum, and penicillin/streptomycin were purchased from GIBCO (Thermo Fisher Scientific, Waltham, MA, USA). Anti-phospho-Akt, anti-total Akt, anti-phospho-Foxo and anti-Foxo antibodies were purchased from Cell Signaling Technology, Inc. (Danvers, MA, USA). Anti-mouse HRP and anti-rabbit HRP were purchased from Thermo Scientific. PVDF membranes and SuperSignal® West Pico and West Femto Chemiluminescent Substrate for western blotting analysis were purchased from Thermo Scientific, and X-ray films from AGFA (Mortsel, Belgium). SYBR Green Master Mix for the real-time PCR analysis was purchased from Applied Biosystems (Foster City, CA, USA).

### Cell culture

The mouse muscle cell line C2C12 (mouse myoblast cell line) was obtained from ATCC (American Type Culture Collection, Manassas, VA, USA). C2C12 cells were cultured in DMEM supplemented with 10% fetal bovine serum and 1% penicillin/streptomycin, at 37 °C, and in 5% CO_2_ and 95% humidified air. Before treatment of KME, C2C12 cells were moved to six-well plates and incubated overnight. Cells were then cultured, with the medium containing 2% horse serum, and incubated for 64 h to induce differentiation of the C2C12 cells. After differentiation, KME (100 μg/ml) was treated for 16 h to stimulate cells.

### Animals

Five-week-old, specific-pathogen-free, male ICR mice were purchased from Dae-Han Laboratory Animal Research Center Co. Ltd (Seoul, Korea). Mice were cared for in the Laboratory of Animal Experiment, Institute of Bioscience, Handong Global University (Pohang, Korea). Mice were maintained in single cages with 12 h light–12 h dark cycles, and were used after 7 days had passed since their arrival. All of the animal experiments were approved by the Ethics Review Committee of the Handong Global University.

### Preparation of cell lysate and western blot analysis

C2C12 cells were prepared for western blotting to detect phospho-Akt, total Akt, and β-actin expression. The cells were washed with cold PBS twice before harvesting. Cells were harvested in 150 μl of cell lysis buffer purchased from iNTRON Biotechnology Inc. (Daejeon, Korea). After collecting the cells, the samples were boiled for 10 min in 95 °C. After boiling, samples were centrifuged at 13,000 rpm for 10 min at 4 °C. The concentration of collected supernatants was determined by a BCA assay kit (Thermo Scientific).

The protein samples (30 μg) were loaded on the 8–10% polyacrylamide gel and blotted on a PVDF membrane in transfer buffer at 200 mA, 2 h after electrophoresis. The blots were blocked with 10% milk in PBST (0.1% Tween-20 in PBS) for 1 h, at room temperature. The blots were incubated with antibodies specific to phospho-Akt (1:1000), Akt(1:1000), phospho-Foxo(1:1000), Foxo(1:1000) and β-actin (1:2000), overnight at 4 °C. The blots were washed three times using PBST, and then incubated with the horseradish peroxidase-conjugated secondary antibody (1:10000) for 1 h, at room temperature. After incubation, the blots were washed again with PBST. After washing, the blots were detected with SuperSignal West Pico or West Femto Chemiluminescent Substrate, using X-ray film.

### Denervation and KME treatment

Six-week-old, male ICR mice were used for the denervation experiments. The right leg was denervated by cutting the sciatic nerve in middle of the leg under anesthesia. KME (200 mg/kg or 500 mg/kg) was given to the mice twice a day, every day, for 2 weeks, through oral administration. After this period of time, whole body grip strength was determined using a grip strength machine, with tests repeated five times. Mice were sacrificed and the quadricepses of the mice were collected and directly used for the experiments or keep at −80 °C for future use.

### Induction of hypertrophy

For hypertrophy, six-week–old, male ICR mice had access to water and the chow contained KME (0.25% KME extract was included) that was ad libitum for 4 weeks. After the 4 weeks, the whole body grip strength was tested using a grip strength machine, and tests were repeated five times. Mice were then sacrificed and quadricepses were harvested and directly used for the experiments or keep at −80 °C for the future experiments.

### Preparing RNA and protein samples from quadricepses

The isolated quadricepses were used in preparing RNA samples for real-time PCR analysis and protein samples for western blotting analysis. PRO-PREP™ lysis buffer was used for preparing protein samples from the quadricepses: the muscle samples were homogenized in the lysis buffer. After homogenization, the samples were centrifuged at 13,000 rpm for 15 min at 4 °C. The concentrations of samples were determined by a BCA assay kit. Total RNA was extracted using the easy-spin™ RNA extraction kit (iNtRON Biotechnology Inc., Seongnam-si, Gyeonggi-do, Korea). Muscle samples were homogenized in the easy-spin™ RNA extraction buffer and the other procedures were performed according to the manufacturer’s instructions.

### Real-time PCR analysis

Total RNA (1 μg) was used for cDNA synthesizing with SuperScript™ II Reverse Transcriptase (Invitrogen, Carlsbad, CA, USA) and an oligo(dT) primer. Real-time PCR was performed using the 7500 sequence detection system (Applied Biosystems) with SYBR Green Master Mix (Thermo Scientific) and ROX™ Reference Dye (Invitrogen). The amount of each reagent and the PCR condition used was according to the manufacturers’ instructions. Relative mRNA expression levels were determined using the ΔΔCt value. β-Actin was used for the endogenous loading control. Experiments were performed with specific primer sequences (Table 1).

**Table 1:** Real-time PCR primer sequences

Gene	Orientation	Primer sequence(5′ → 3′)
PGC-1α	Forward	TCGATGTGTCGCCTTCTTGC
	Reverse	ACGAGAGCGCATCCTTTGG
Atrogin-1	Forward	ATTCTACACTGGCAGCAGCA
	Reverse	TCAGCCTCTGCATGATGTTC
Murf1	Forward	ACCTGCTGGTGGAAAACATC
	Reverse	AGGAGCAAGTAGGCACCTCA
GLUT4	Forward	AACCAGCATCTTCGAGTCGG
	Reverse	CGAGACCAACGTGAAGACCG
SREBP-1c	Forward	GGAGCCATGGATTGCACATT
	Reverse	GGCCCGGGAAGTCACTGT

### Analysis of skeletal muscle fiber

Skeletal muscle tissues (quadriceps) were fixed overnight in 4% formaldehyde at room temperature. Then, skeletal muscle samples were frozen in optimal cutting temperature (OCT) and sectioned at 5 μm for hematoxylin and eosin (H&E) staining. Images were captured with Motic BA300 microscope and ImageJ software (developed by Wayne Rasband, National Institute of Health, Bethesda, MD) was used for analysis.

### Statistical analysis

Figures were expressed using GraphPad prism 5 software (GraphPad Software, Inc., CA, USA). Data were presented as mean ± S.D. and analyzed by one-way ANOVA *t*-test. P values < 0.05 were considered as significant.

## Results

### KME treatment induces the activation of the PI3K/Akt pathway in cells

**Fig. 1:**
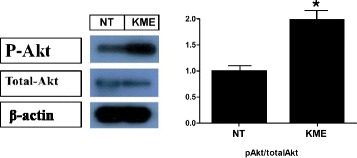
Activation of the Akt signaling pathway by KME (*Left*) Relative phosphorylation of Akt (P-Akt) is shown. The concentration of KME was 100 μg/ml. (*Righ*t) The relative amount of phospho-Akt is shown. All the samples were blotted against β-actin as a loading control. NT: non-treated cells. P-values of < 0.05 and < 0.01 are indicated by * and **, respectively

To investigate the muscle atrophy inhibition and muscle hypertrophy stimulation by KME, we first checked whether the PI3K/Akt pathway is activated in C2C12 cell lines. KME treated cells, and a non-treated control cells were also established. In the KME-treated cells, higher phosphorylation of Akt was detected compared with non-treated control cells (Fig. 1). These data suggest that KME has an effect on the regulation of the muscle mass through the activation of the Akt/mTOR signaling pathway.

### KME treatment regulates other metabolic pathways in cells

**Fig. 2:**
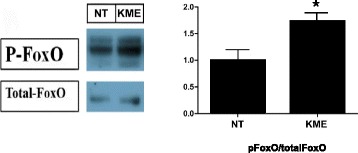
The effect of KME on the phosphorylation of FoxO. Phosphorylation of FoxO (P-FoxO) by KME(100 μg/ml) treatment in C2C12 cells. (*Right*) The relative amount of phospho-FoxO is indicated. P-values of < 0.05 and < 0.01 are indicated by * and **, respectively

To investigate the effect of KME on muscle mass regulation further, we also examined the phosphorylation of FoxO in C2C12 cells. FoxO is a known key molecule of muscle atrophy due to its role in the activation of protein degradation [13]: FoxO induces muscle atrophy by stimulating the E3 ubiquitin ligases Murf1 and Atrogin-1 [26]. Compared to the non-treated control cells, the KME-treated cells showed increased phosphorylation of FoxO (Fig. 2). These data also support our previous observation that KME could induce the phosphorylation of AMPK, which is a repressor of FoxO.

### KME treatment regulates muscle atrophy related gene expression in cells

**Fig. 3:**
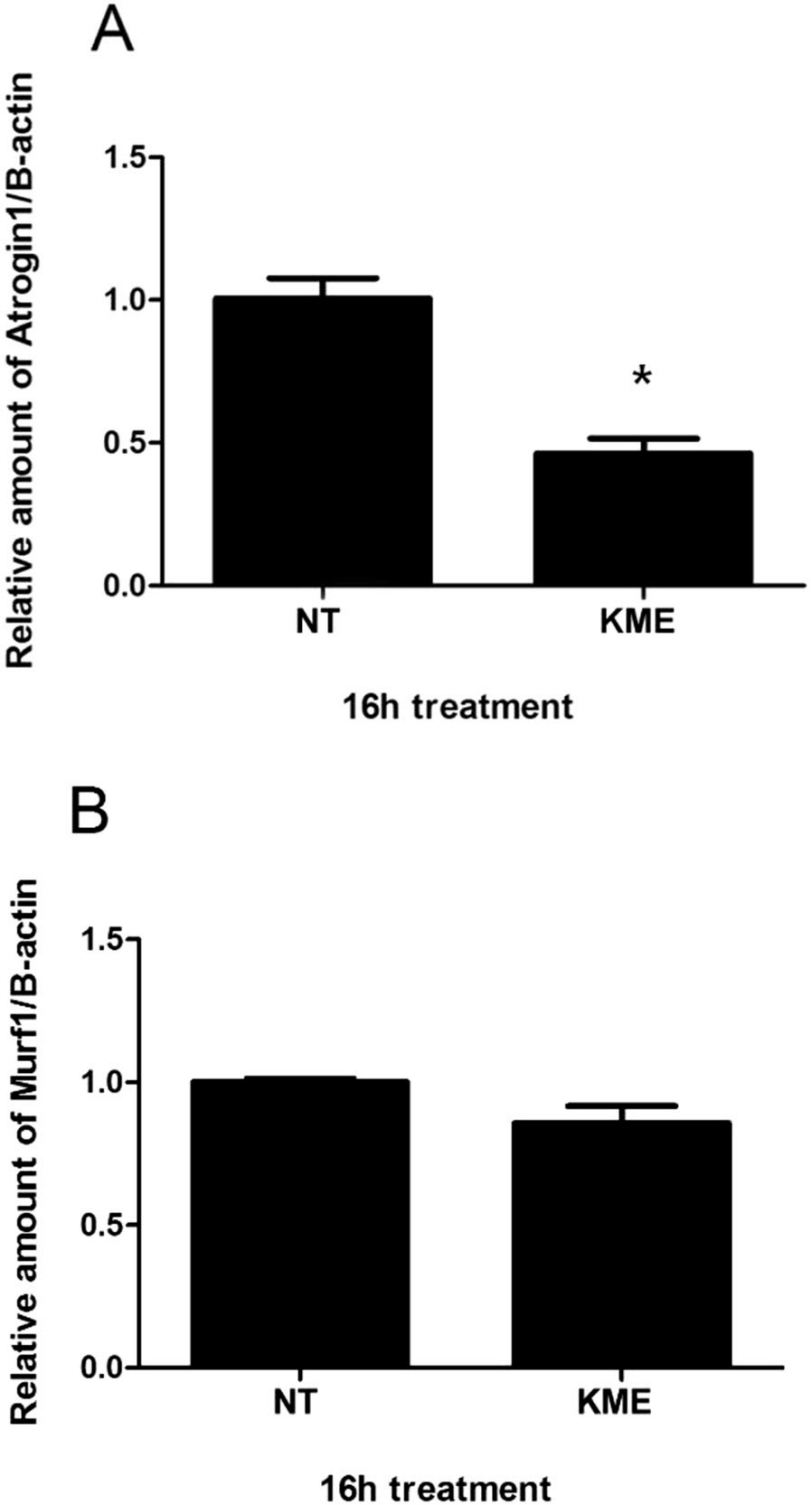
The inhibition of muscle atrophy related gene expression by KME (**a**) The relative amount of Atrogin-1 was measured after KME treatment. The final concentration of KME was 100 μg/ml. **b** The relative amount of Murf1 is shown. The mRNA expression levels were compared to β-actin. Data are shown as means of the relative expression levels ± SEM. P-values of < 0.05 and < 0.01 are indicated by * and **, respectively

**Fig. 4:**
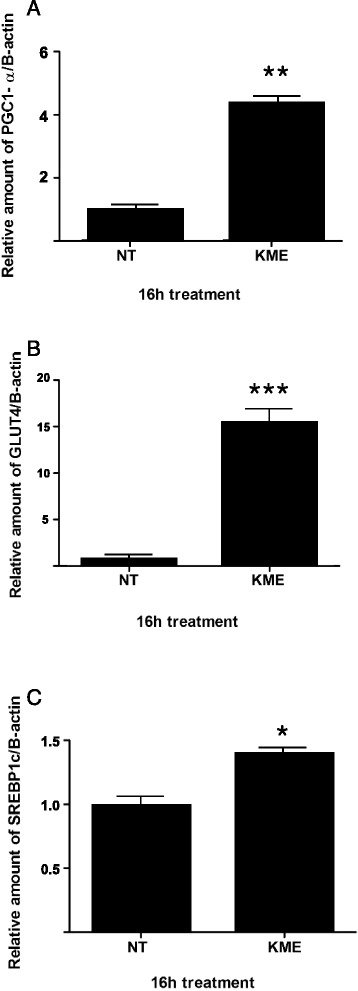
The induction of muscle atrophy and hypertrophy related gene expression by KME. **a** The relative amount of PGC-1α is shown. **b** The relative amount of GLUT4 is shown. **c** The relative amount of SREBP-1c is shown. All the analyses were performed after treatment of KME at a final concentration of 100 μg/ml. The mRNA expression levels were compared to β-actin. Data are shown as means of the relative expression levels ± SEM. P-values of < 0.05 and < 0.01 are indicated by * and **, respectively

To determine the direct effect of KME in the regulation of muscle atrophy, we checked the mRNA expression of genes related to muscle atrophy in C2C12 cells. A real-time PCR method was used to measure the expression levels of Murf1 and Atrogin-1, so-called “atrogenes”. In addition, the mRNA expression levels of PGC-1α, GLUT4, and SREBP-1c, that regulate the expression of Atrogin-1 and Murf1, were also examined. After 16 h, the KME-treated group showed a decreased gene expression of Atrogin-1, compared to the non-treated group (Fig. 3). On the contrary, the KME-treated group showed increased mRNA expression of PGC-1α, GLUT4, and SREBP-1c, compared to the non-treated group (Fig. 4). These genes are related to the inhibition of muscle atrophy and are related to the induction of muscle hypertrophy [27–29]. These analyses demonstrate the multifunctional effects of KME in the activation of Akt and AMPK signaling pathways.

### KME lessens the decrease of muscle mass in denervated mouse models

**Fig. 5:**
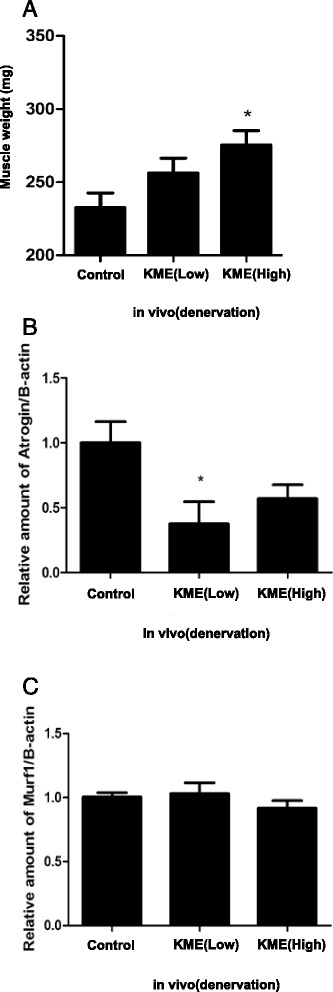
Decreased muscle atrophy in KME-fed denervated mice (**a**) The weight of quadricepses is shown. **b** The relative amount of Atrogin-1 is shown. **c** The relative amount of Murf1 is shown. Data are shown as means of the relative expression levels ± SEM; n = 5 per group. P-values of < 0.05 and < 0.01 are indicate by * and **, respectively. KME(low) : 0.3%, KME(high) : 1.5%

Based on our data from real-time RT-PCR in C2C12 cells, we hypothesized that KME might reduce muscle atrophy in vivo, using the animal model. To validate this hypothesis, we tried to selectively denervate the right quadricepses of mice by amputating each right leg’s sciatic nerve. Denervation is a common method to induce artificial muscle atrophy in the specific skeletal muscle. In our study, we designated control group the PBS-treated denervated group. After denervation, we treated KME (200 mg/kg and 500 mg/kg) to the mice every 12 h by oral gavage for 15 days. After 15 days of treatment, KME treated mice showed decreased denervation induced by muscle atrophy (Fig. 5a). In the mRNA expression levels monitored, Murf1 expression was not affected by KME treatment. However, in the case of Atrogin-1, KME (low fed) decreased the expression of Atrogin-1 compared to the KME non-treated group (Fig. 5b and c). These data suggest that KME might repress the artificial muscle decrease, and this effect is closely related to the regulation of Atrogin-1 gene expression.

### KME increases muscle mass and grip strength in native conditions

**Fig. 6:**
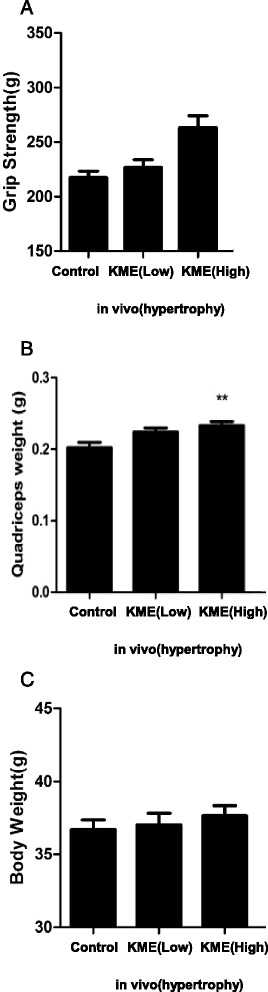
Physical changes in KME-fed mice (**a**) The grip strength of each chow-fed mouse is presented. **b** The weights of quadricepses are shown. **c** Whole body weights are shown. All the mice were fed chow for 4 weeks and analyses were done after the 4 weeks. Data shown are mean ± S.D.; n = 5 per group. P-values of < 0.05 and < 0.01 are indicate by * and **, respectively. KME(low) : 0.3%, KME(high) : 1.5%

The reduction of muscle atrophy by KME led us to hypothesize that KME might induce muscle hypertrophy. This hypothesis is based on the observation that KME induces the upregulation of muscle atrophy related genes and the activation of the Akt signaling pathway in C2C12 cells. To evaluate the muscle hypertrophic effects of KME in vivo, mice were allowed free access to control chow or chow containing 0.3% or 1.5% KME for 4 weeks. Compared to the control mice, the KME-containing chow-fed mice showed increased whole body weights, a higher weight of quadricepses, and increased grip strengths in high KME-fed group (Fig. 6). These data present the morphological indication that KME might induce skeletal muscle hypertrophy in mice.

### KME showed increased endurance in vivo

**Fig. 7:**
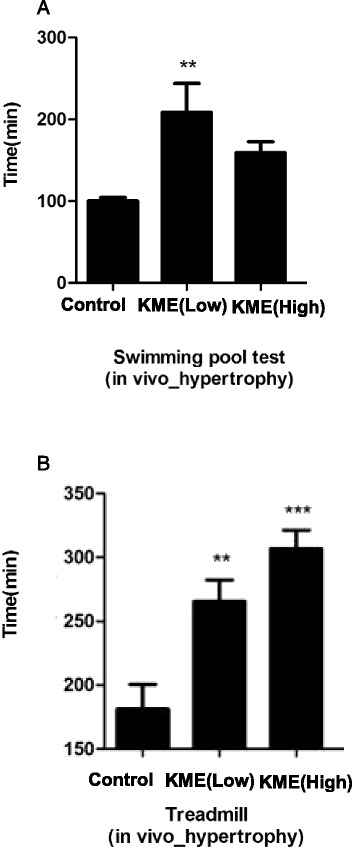
The increased endurance in the KME-fed mice (**a**) The endurance results of the mice measured by swimming pool tests are shown. **b** The endurance results of the mice measured by the treadmill tests are shown. All the mice were fed chow for 4 weeks**.** Data signifies means ± S.D.; n = 5 per group. P-values of < 0.05 and < 0.01 are indicate by * and **, respectively. KME(low) : 0.3%, KME(high) : 1.5%

**Fig. 8:**
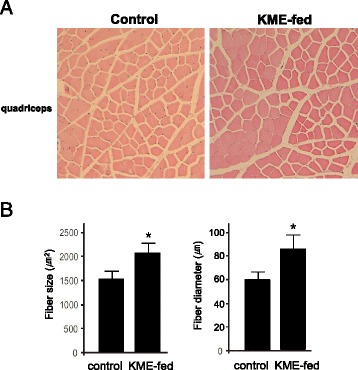
The increased muscle fiber size and diameter in the KME-fed(1.5%) mice (**a**) Cross-sections of quadriceps muscle stained with H & E. **b** Fiber size and diameters were measured from quadriceps muscle sections from a total of 500 fibers (n = 5 mice per group). All the mice were fed chow for 4 weeks**.** Data signifies means ± S.D.; P-values of < 0.05 is indicate by *

To validate the muscle hypertrophic effect of KME, the treadmill and swimming pool tests were performed after the feeding of control chow or KME-containing chow over the 4 weeks. KME-containing chow-fed mice showed increased swimming activity and elevated running times on the treadmill compared to the control chow-fed mice (Fig. **7**). In addition, skeletal muscle area and diameter were also increased in KME-fed mice (Fig. **8**).

## Discussion

Previous reports regarding muscle atrophy and hypertrophy have shown that muscle atrophy induced by various kinds of mechanisms—such as fasting, illness (cancer and diabetes), and muscle disuse—all result in similar specific increases of mRNA expression [30, 31]. These similar specific genes are called “atrogenes” [32]. In this present study, we showed that these genes (Murf1 and Atrogin-1) are downregulated by KME treatment, suggesting its effect on the inhibition of muscle atrophy. Atrogin-1 is known to promote the degradation of MyoD and elF3f [33]: MyoD is an important transcription factor of muscle and elF3f is one of the enhancer proteins of protein synthesis [34, 35]. Atrogin-1 degrades both of these proteins resulting in muscle atrophy. Murf1 regulates muscle structural proteins like troponin 1 and myosin [11, 36].

Mistletoe extracts were already used in broad spectrums as remedying agents [37, 38]. Based on the activation of the Akt signaling pathway and AMPK, we speculated that mistletoe extract could regulate cellular signaling involved in the regulation of muscle mass because these pathways are closely related to muscle atrophy and hypertrophy. Consistent with our prediction, we found that KME inhibits “atrogene” expression in C2C12 cells. Thus, we adopted an in vivo animal model to validate our observation in myotubes. In a subsequent study, we used a well-known method to induce muscle atrophy called denervation [39, 40]. After denervation of the sciatic nerves of the control mice, Murf1 and Atrogin-1 mRNA expression were increased, and the muscle mass were decreased. Grip strength was also reduced in the control mice. Compared with these control mice, KME treated mice showed a decreased expression of Atrogin-1 and Murf1, increased muscle mass, and greater grip strength.

We also investigated other signaling molecules involved in muscle atrophy repression and muscle hypertrophy in KME-treated cells and mice. However, there were no significant differences in the phosphorylation of mTOR and S6k by KME [41, 42]. Recently, it we reported that mTOR promotes muscle atrophy in denervated mice through the activation of FoxO and E3 ubiquitin ligases [43]. This is exactly opposite to the current paradigm depicting the function of mTOR in muscle hypertrophy and muscle atrophy [44]. The current dogma of mTOR functionality is the stimulation of muscle hypertrophy in skeletal muscle [45]. Therefore, there are still controversies about the role of mTOR in muscle physiology. Further studies are needed to figure out the relationship between the mTOR signaling pathway and the induction of muscle atrophy.

Another well-known signaling pathway involved in muscle atrophy is the NF-κB one [46]. This signaling pathway is also closely related with inflammation [47]. It was also reported that inflammation was repressed by mistletoe extract [48]. Further studies are required to elucidate whether KME treatment affects inflammation and NF-κB signaling in the inhibition of muscle atrophy.

Extending from the induced muscle atrophy model investigated, we also tried to test whether KME induce muscle hypertrophy in mice skeletal muscle. It is proven that an inhibition of muscle atrophy is associated with muscle hypertrophy [7]. We also proved this kind of hypertrophic effect with KME treatment in this study. Without any artificial induction of muscle atrophy, mice that fed on KME for 4 weeks showed increased muscle mass and grip strength. These results suggest that KME affects not only muscle atrophy but also muscle hypertrophy. Furthermore, our results also imply that KME can regulate both atrophy and hypertrophy independently. It was confirmed by the endurance tests that KME regulates muscle hypertrophy independent of muscle atrophy [49]. KME-containing chow-fed mice showed increased endurance compared to the control chow-fed mice. Muscle hypertrophy and increased endurance were closely related with the muscle fiber type switching [50, 51]. It is noteworthy that the study on the type switching of the muscle fiber might give us another molecular clue of the effects of KME in muscle regulation. In addition, a comparative study on the effects of KME in cardiac muscle and skeletal muscle is required—several reports demonstrated that the function of the mTOR signaling pathway is different between skeletal muscle and cardiac muscle [52].

In conclusion, the results of this study suggest that KME has positive regulatory effects on the maintenance of muscle mass. Our data shows the possibilities that KME could be used as a potential therapy for muscle atrophy in human cases.

## Conclusions

Korean mistletoe extracts have inhibitory effects on muscle atrophy and stimulatory effects on muscle hypertrophy in C2C12 cells and denervated mice. These findings suggest that Korean mistletoe might be a potential candidate as a functional food promoting the restoration from muscle atrophy.

## Abbreviations

ATCC: American Type Culture Collection
DMEM: Dulbecco’s modified Eagle’s medium
FBS: Fetal bovine serum
KME: Korean mistletoe extract
MAPK: Mitogen-activated protein kinase
NC: Nitrocellulose
PBS: Phosphate-buffered saline.
